# Algorithmic matching of personal protective equipment donations with healthcare facilities during the COVID-19 pandemic

**DOI:** 10.1038/s41746-020-00375-3

**Published:** 2021-01-29

**Authors:** Ram Bala, Charlotte Lee, Benjamin Pallant, Maahika Srinivasan, Daniel Lurie, Rohit Jacob, Neeraj Bhagchandani, Megan Ranney, Shuhan He

**Affiliations:** 1 Department of Information Systems and Analytics, Santa Clara University, Santa Clara, CA USA; 2 Warren Alpert Medical School, Brown University, Providence, RI USA; 3 Harvard Medical School, Boston, MA USA; 4 Department of Psychology, University of California, Berkeley, USA; 5 Brown-Lifespan Center for Digital Health, Alpert Medical School, Brown University, Providence, RI USA; 6 Center for Innovation in Digital HealthCare, Lab of Computer Science, Department of Emergency Medicine, Massachusetts General Hospital, Harvard University, Boston, MA USA

**Keywords:** Health care economics, Health policy, Health occupations

## Abstract

GetUsPPE.org has built a centralized platform to facilitate matches for PPE donations, with an active role in matching donors with the appropriate recipients. A manual match process was limited by volunteer hours, thus we developed an open-access matching algorithm using a linear programming-based transportation model. From April 14, 2020 to April 27, 2020, the algorithm was used to match 83,136 items of PPE to 135 healthcare facilities in need across the United States with a median of 214.3 miles traveled, 100% of available donations matched, met the full quantity of requested PPE for 67% of recipients matched, and with 46% matches under 30 miles traveled. Compared with the period April 1, 2020 to April 13, 2020, when PPE matching was manual, the algorithm resulted in a 280% increase in matches/day. This publicly available automated algorithm could be deployed in future situations when the healthcare supply chain is insufficient.

## Introduction

The arrival of Coronavirus Disease 2019 (COVID-19, caused by the novel virus SARS-CoV-2) in the United States and subsequent reports of community transmission in states including Washington and California greatly increased awareness of potential medical supply shortages for responding to a widespread respiratory disease outbreak. Early concerns about inadequate ventilators and hospital/ICU beds necessary to handle a surge of patients^1,2^ were quickly joined by reports of significant global shortages of the personal protective equipment (PPE) used to minimize frontline healthcare workers’ risk of becoming infected^3–6^. The risk to healthcare professionals (HCPs) of infection with COVID-19 appears significant^7^, with HCPs reflecting over 11% (3,155 of 27,528) of all cases confirmed as of April 16, 2020 in the state of California^8^; similar rates have been reported in other states that document whether confirmed positive cases were among HCPs^9^. The PPE shortage was met with a multifaceted response. The US Food and Drug Administration (FDA) issued an Emergency Use Authorization (EUA) expanding the quality and approval standards for filtering facepiece respirators (e.g., N95s) allowed for use in medical efforts during the pandemic^10^. A variety of private-sector manufacturers expanded or redirected their efforts to produce medical supplies, in some cases under mandate of the Defense Production Act^11^. In addition, numerous grassroots efforts arose to respond to the need. Organizations with 3D-printing and related fabrication capabilities began producing open-source models of PPE with guidance from the FDA^12–15^. Other groups formed at both local and national scales to help facilitate and match PPE donations from individuals, small business, and other private donors to medical facilities in need^16–18^. Originally formed as a coalition through the viral Twitter hashtags #GetUsPPE and #GetMePPE, GetUsPPE.org quickly became a nationwide organization focused on a mission to “build a national, centralized platform to enable communities to get PPE to healthcare providers on the frontlines of the COVID-19 pandemic”^19^. The original platform specification empowered donors with smaller quantities of PPE (e.g., several boxes of gloves, 3–4 packaged N95s) to facilitate their own donations by selecting an organization in need from a map that populated potential recipient organizations from the database. These requirements quickly changed due to the need for recipient organizations’ information to be protected, given large amounts of PPE-related scams and reports of retribution by healthcare organizations toward employees who raised concerns about PPE shortages^20–22^. Additionally, the wide variation among donations in terms of quantity, item type, and geography demonstrated a need for the organization to assume an active role in matching donors with the appropriate recipient.

GetUsPPE assembled a volunteer team to facilitate connections between donors and local recipients. This required a volunteer to segment lists of both donors and recipient sites by geographic region (most often, within a particular state), confirm that suppliers still had inventory available for donation via phone/email, search for a recipient organization in the database that both requested the items available and is in reasonable (subject to the volunteer’s judgment) vicinity of the donor, possible by volunteer transport. The volunteer confirmed recipient need and acceptance of supplier quality and then linked the donor and a possible recipient via email to confirm delivery location and time. The match process often necessitated a third-party transportation volunteer if the donor was unable to get the supplies to the recipient independently. This challenge was made more complex by trying to maximize the amount of need that could be met while minimizing or eliminating the financial and time costs of shipping or independent transportation for either donors or GetUsPPE.org volunteers. Efforts to manually match donors to recipients was not a scalable solution, with donations and requests substantially outpacing volunteer capacity to match and subsequently process them.

GetUsPPE and our collaborators aimed to identify ways in which the donor-to-recipient matching process could be automated, allowing for faster matching of larger sets of supply and demand data. In particular, this manuscript documents the development of an open-access matching algorithm to optimize PPE donation and request matches. The algorithm is based primarily on PPE quantities offered and requested and geographic proximity as initial variables to maximize the amount of need met while minimizing delivery miles for GetUsPPE and its donors and partners.

## Results

### Match descriptive statistics

From April 14, 2020 to April 27, 2020, we used the PPE-matching algorithm to match 83,136 items of PPE to 135 healthcare facilities in need across the United States (Table 1). Of these 135 donor–recipient matches, hospitals made up the largest proportion of healthcare facility recipients (27%), with surgical masks making up most of the matched donations (65%). Matched donation sizes varied, with the largest proportion being between 100 and 500 items (27%), followed by small donations of 10–50 and 0–10 items (both 24%).

**Table 1:** Match characteristics.

	*n* (Number of donor–recipient matches)	%
Facility type		
Hospital	36	26.7
Outpatient facility	28	20.7
Nursing home/assisted living facility	16	11.9
Federally qualified health center	15	11.1
Home health/visiting nursing services	9	5.9
Hospice services	8	6.6
Skilled nursing facility/rehabilitation facility	7	5.2
Lab/diagnostic center	4	3.0
Inpatient psychiatric facility	4	3.0
Emergency medical services	4	3.0
Urgent care	3	2.2
Organ procurement organization	1	0.7
Total	135	100
Donation size		
0–10	32	23.7
10–50	32	23.7
50–100	13	9.6
100–500	36	26.7
500–1000	9	6.7
1000–2000	2	1.5
2000+	11	8.1
Total	135	100
PPE types		
Surgical masks	88	65.2
KN95s/N95s	16	11.9
Face shields	10	7.4
Body suits/coveralls	9	6.7
Gloves	10	7.4
Booties	1	0.7
HEPA respirator	1	0.7
Total	135	100

### Algorithm effectiveness

PPE donor–recipient matches had a median distanced traveled of 214.3 miles. All available donations were used (100% of supply). The algorithm distribution met the full quantity of requested PPE for 67% of recipients matched (Table 2). Nearly half of matches traveled under 30 miles (46%) from donors to healthcare facility recipients.

**Table 2:** Algorithm effectiveness.

	*n* (Number of donor–recipient matches)	%
Distance traveled		
0–15 miles	29	21.5
15–30 miles	33	24.4
30–50 miles	41	30.4
50–100 miles	4	3.0
100 miles +	28	20.7
Total	135	100
Matched recipients		
Partial need met	91	32.6
Full need met	44	67.4
Total	135	100

**Fig. 1:**
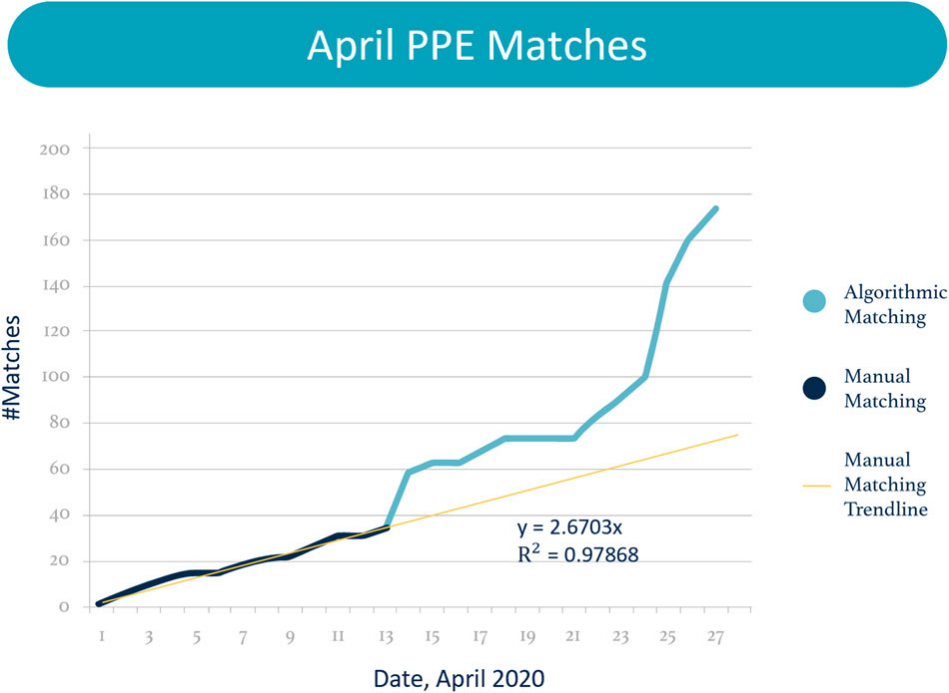
April PPE matches. Algorithmic matching began on April 14.

Prior to introduction of the matching algorithm on April 14, volunteers manually matched 34 donors with recipients. From April 14 to April 27, after the matching algorithm was introduced, an additional 139 matches were made (135 were made by the algorithm and 4 were made manually) (Fig. 1). Each individual donor-to-recipient match is logged as one match regardless of the number of pieces of PPE, which ranged from 1 to >2000, depending on the nature of supply and demand. Prior to the algorithm, matches were made using a laborious manual method where volunteers have to determine the distance between potential donors and recipients and use their best judgment to balance supply and demand. Use of the algorithm converts matching into a batch process where a larger subset of donors and recipients are fed into the algorithm, which then performs matches based the optimization model described earlier, within a minute. However, matches are recorded as completed only when volunteers verify that the donor for a match is still able to ship the donation as originally indicated. This is reflected in Fig. 1 where the number of matches rises gradually rather than in a step fashion despite the use of a batch algorithm. However, several characteristics of this graph pre-algorithm and post-algorithm deployment merit attention.

### Impact of the algorithm on speed of matching

**Fig. 2:**
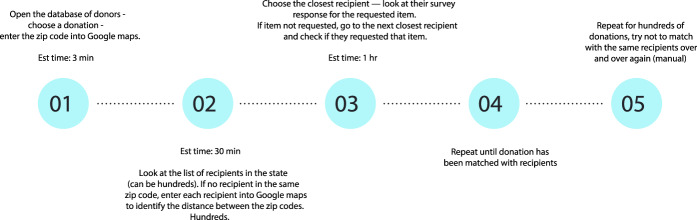
Manual matching process description. A manual match process was limited by volunteer hours.

The authors of the manuscript attempted to identify the volunteer hours early in the process to quantify the efficiency of the match, but due to the rapid turnover of the volunteers as well as the heterogeneity in how long each match took, we determined it was not feasible. However, in order to better illustrate the process time and the difficulty, we have created Fig. 2 to better demonstrate how difficult this task was. Prior to the initiation of the algorithm between the dates of April 1, 2020 and April 14, 2020, we initiated 249 emails that were sent to donors in order to inquire about the availability of their stated supply and facilitate a match. Of those 249, only 23 initiated contacts resulted in a confirmed, executed match. The median number of emails exchanged with a single donor in order to facilitate a successful match in this period was 6—the number of emails in a chain that one volunteer would be managing in order to carry through a match.

After implementation of the algorithm from April 14, 2020 to April 27, 2020, our study period, we sent out 62 emails to donors. The algorithm could match multiple recipients with a single donor, thus one donor email could initiate multiple matches. 14 potential donors stated they had no supply left, resulting in 138 successful matches during the period. The median number of emails exchanged with a single donor to facilitate a successful match was 7. This is slightly higher than the pre-algorithm phase but is a justifiable increase in light of the higher quality of matches. Each donor’s supply may be split across multiple recipients to better match supply and demand, a key benefit of the algorithm. Table 3 summarizes these details.

**Table 3:** Donor e-mail outreach statistics.

	Emails initiated	No. of supply left	Successful matches
Apr.1–Apr.14 (control)	249	49	23
Apr.14–Apr. 27 (study)	62	14	138
% Change	−75.1%	−71.4%	+600%

As per Fig. 1, we see linear growth rate in the number of matches prior to use of the algorithm. A linear regression fit with *R*^2^ = 0.98 shows strong support for this. Matches occur at the rate of approximately 2.67 matches per day (slope of the linear graph). A straight average of pre-algorithm matches yields 2.62 matches per day on average. Supply on the GetUsPPE Platform is growing but is volatile. The strict linear relationship between the number of matches and time during the manual matching phase despite volatile supply indicates a system with a matching capacity (measured in volunteer hours) bottleneck^23^. Although one could add more volunteers to speed up the manual matching process, automated matching speeds up the process substantially and the the number of matches begins to track supply much more than earlier, revealing that matching capacity is no longer a bottleneck. The extent of growth in matching due to the algorithm is visually captured in the graph as the gap between the extrapolated linear trendline of manual matches and the actual the number matches from April 14. Specifically, after the algorithm was employed, there was a 280% increase in the number of matches made per day (Table 4). Use and iterative improvement of the algorithm is continuing through GetUsPPE in response to the ongoing COVID-19 pandemic.

**Table 4:** Pre/Post Algorithm Matches.

	Pre-algorithm	Post-algorithm	% Change
Matches/day	2.62	9.93	279.6%

## Discussion

The PPE-matching algorithm described above efficiently allocates PPE donations to healthcare facilities and has allowed GetUsPPE to scale up PPE donation coordination efforts significantly. Specifically, the advantages of the algorithm are two-fold:

1. Automation: proximity measurement is automated using appropriate distance functions that convert zip codes into latitude and longitude coordinates. A distance-based sorting algorithm can be applied once a distance matrix is calculated quickly.
2. Minimizing supply–demand mismatches: this ensures that no PPE is wasted at a healthcare facility. This is very difficult to implement manually even for 10 donors and 10 recipients. In the algorithm, this is achieved by the use of binding constraints on supply to individual facilities in an optimization problem. The binding constraint ensures that no healthcare facility gets more than what is asked for. This binding constraint is relaxed in post-processing only for small donors (below a user-specified threshold) if the donor’s supply cannot be split across recipients.

The algorithm relies on PPE demand that is self-reported by healthcare facility representatives and PPE supply that is voluntarily reported by individuals or organizations for donation through GetUsPPE.org. Using these inputs, the algorithm maximizes the demand met while minimizing the shipment-miles-traveled, which results in lower shipping costs, shorter volunteer drives for drop-offs, and faster distribution.

We were unable to analyze for the significance of the difference in pre-algorithm and post-algorithm matches/day due to confounding factors, including continued onboarding of additional volunteers and improved workflows. While the algorithm takes distance, PPE type, and quantity into consideration, it does not currently account for geographic and facility-level variations in COVID-19 prevalence, which would likely influence ongoing PPE needs. Additionally, this initial matching algorithm does not inherently prioritize any organization over the other, preventing us from factoring in equity and bioethical concerns such as high urgency of need (i.e., PPE out of stock) or organizations that serve particularly vulnerable populations (skilled nursing facilities, Indian Health Services, or rural safety net hospitals). Further, the algorithm is unable to account for organizations that have not self-reported demand to the public GetUsPPE website; the geographic distribution of demand input data inevitably affects the allocation of PPE supply. To address these limitations, moving forward, we are performing targeted outreach to increase the GetUsPPE supply and demand data, pursuing partnerships with public health and bioethics experts to address equity concerns in distribution, and collaborating with epidemiology and predictive modeling experts to better account for COVID-19 patterns of disease spread.

The ability to use a PPE-matching algorithm to connect PPE donors and recipients en masse has significant ramifications for controlling the spread of COVID-19 and preserving the healthcare workforce. Given that the global stockpile of PPE was inadequate prior to the emergence of COVID-19, GetUsPPE.org’s role in developing an automated algorithm to efficiently match the existing donated supply of PPE to nearby recipients serves as a critical stopgap to break the chain of transmission, while new global PPE production pipelines and supply chains are established. This publicly available matching algorithm has implications for emergency supply chains beyond the world of PPE. In any humanitarian context, that is characterized by fragmentation of supply and demand quantities, significant geographic dispersion in those supply / demand points and fluctuations in these points as a function of time, is a potential candidate for large scale application of this algorithm at a regular cadence. Such scenarios are widespread across delivery of medical supplies and food to vulnerable populations^24–27^. One limitation of the current software is that usage is restricted to the continental United States. Further research and development is needed to expand the geographic reach of this open-source algorithm.

## Methods

### Data collection

**Fig. 3:**
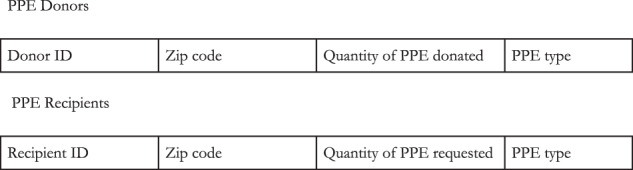
Algorithm input data. Data collected from individuals offering to donate PPE “donors” and healthcare facilities requesting PPE “recipients".

**Fig. 4:**
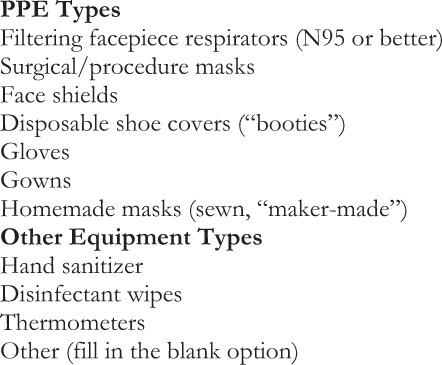
PPE and equipment types. Recipients were able to request multiple types of PPE.

Input data (Fig. 3) was collected from individuals offering to donate PPE, “donors,” and healthcare facilities requesting PPE, “recipients,” for analysis and matching by GetUsPPE.org. Recipients were able to request multiple types of PPE (Fig. 4) and asked not to request more than 1 week’s supply of each type of PPE.

### Matching algorithm

The data collected by GetUsPPE.org was input into an open-source matching algorithm. The key objectives that this matching allocation satisfies are:

1. Minimizes the shipment-miles-traveled in the network (miles traveled weighted by shipments)
2. Balances supply and demand with a few specific conditions:

- All supply is exhausted if demand exceeds supply.
- If supply exceeds demand (a rare event in this scenario), all demand is met by minimizing shipment miles and excess supply is held back for future requests.
- Recipients may get less than what they ask for if demand exceeds supply (which is typical). This is measured by the metric called fill rate (Eq. 1)^28^.

1 $${\mathrm{Fill}}\;{\mathrm{rate}}\;{\mathrm{of}}\;{\mathrm{recipient}} = \frac{{{\mathrm{No.}} \;{\mathrm{of}}\;{\mathrm{units}}\;{\mathrm{of}}\;{\mathrm{PPE}}\;{\mathrm{supplied}}\;{\mathrm{to}}\;{\mathrm{recipient}}}}{{{\mathrm{No.}} \;{\mathrm{of}}\;{\mathrm{units}}\;{\mathrm{of}}\;{\mathrm{PPE}}\;{\mathrm{requested}}\;{\mathrm{by}}\;{\mathrm{recipient}}}}$$

3. Minimize logistics complexity for small donors by precluding multiple shipments from the donor. “Small” donor is defined by a parameter on donor capacity. For example, if this parameter is 50 for masks, then a donor with 50 or fewer masks to donate will never be asked to ship to multiple recipients. This parameter is defined by the match volunteers, based on product type and fill rate considerations. While the “small” donor threshold for gowns might be 50, for gloves that threshold might be better set at 200 due to packaging volume.

The construction of the objective function and the constraints followed best practice in the design of matching markets^29,30^. In our experience, the choice of distance traveled per unit of PPE as a key element of the objective function mattered for several reasons:

1. Donors often prefer donating locally.
2. Donors are more likely to deliver their PPE supply on their own if the recipient is closer.
3. If the donor is unwilling to deliver, finding a volunteer for the same activity is easier if the recipient is closer.
4. If neither donor nor volunteer is available, GetUsPPE ships through its logistics partners. The logistics is funded through $ donations by financial donors, which is limited. Therefore, minimizing distance while maximizing impact is a core goal of the organization in order to stay within allocated budgets. Every $ saved on logistics can be used to buy scarce PPE and other resources required for the organization’s primary mission.

The code is written in Python and follows several steps as described below:

Step 1: We use the distance function “Haversine” to compute the distance between every donor zip code and every recipient zip code. For example, if we have 3 donors and 2 recipients, there will be 3*2 = 6 possible distances. This distance function converts every zip code into a latitude and longitude specification. Distance between any two zip codes (measured by latitude / longitude) can be computed using basic coordinate geometry. We use the notion of Haversine distance, a generalized form of Euclidean distance^29^, which is also referred to in layman’s terms as “as the crow flies”. However, unlike Euclidean distance, Haversine distance incorporates the shape of the Earth in the computation of distance.

Step 2: We create a unique identifier for each donor and recipient and assign the above distances to a distance matrix marked by these identifiers. We also ingest supply capacity and donor request into supply and demand arrays marked by these identifiers.

Step 3: We calculate total supply by adding all donor capacity and total demand by adding all donor requests. If total supply is less than total demand, we leave supply and demand arrays as it is. If total supply exceeds total demand, we create a dummy recipient with a large demand value (for example, a million). We assign a large distance (for example, use Earth’s South Pole as the location of this recipient) between this recipient and every donor in our donor pool.

Step 4: We use Google Linear Optimization^30^, an open-source Linear Programming Solver to solve the optimization problem as defined below:

The mode is specified for an individual PPE type but is repeatable across each type:

Notation:

Let the donors be indexed by *i* = 1,2,3….

Let the recipients be indexed by *j* = 1,2,3…

Let *X*(*i*,*j*) be the quantity shipped by donor *i* to recipient *j* (this is the variable)

Let *d*(*i*,*j*) be the distance from donor *i* to recipient *j* (this is a parameter)

Let *S*(*i*) be the available supply at donor *i* (parameter)

Let *A*(*j*) be the “ask” at recipient *j* (parameter).

The optimization problem is as described in Eq. 2:

2 $$\displaystyle{\mathrm{Minimize}}\;{\mathrm{over}}\;X(i,j)\;{\mathrm{the}}\;{\mathrm{objective}}\;({\mathrm{shipment}} - {\mathrm{miles}}):\mathop {\sum}\limits_i {\mathop {\sum}\limits_i {d(i,j)} \times X(i,j)}$$

subject to the following constraints as described in Eq. 3:

3 $$\begin{array}{l}\mathop {\sum }\nolimits_i^{\,} X(i,j)\, \le A(j)\,{\mathrm{for}}\;{\mathrm{each}}\;j:{\mathrm{Recipients}}\;{\mathrm{do}}\;{\mathrm{not}}\;{\mathrm{get}}\;{\mathrm{more}}\;{\mathrm{than}}\;{\mathrm{what}}\;{\mathrm{they}}\;{\mathrm{ask}}\;{\mathrm{for}}\\ \mathop {\sum}\nolimits_j {X(i,j) = S(i)\,{\mathrm{for}}\;{\mathrm{each}}\;i:{\mathrm{All}}\;{\mathrm{donor}}\;{\mathrm{supply}}\;{\mathrm{is}}\;{\mathrm{exhausted}}} \end{array}$$

Step 5: We filter out all *X*(*i*,*j*) values >0. These are the baseline shipment allocations and a positive match between Donor *i* and Recipient *j*. If total supply exceeds total demand (measured in Step 3), then any (*i*,*j*) match where recipient *j* is the dummy recipient is excluded from the matching set. Donors *i* who remain unmatched are flagged and potentially carried forward to a future allocation.

Step 6: We filter out each donor *i* for whom more than 1 match (*i*,*j*) is >0 and donor supply *A*(*i*)≤ parameter specified by user (default = 50). For each donor *i* identified by this process, we sort *X*(*i*,*j*) values in descending order; define *X*(*i*,*j*)max as the highest of these values. In case of a tie, we pick the highest *X*(*i*,*j*) with a lower distance *d*(*i*,*j*). In case of a tie on distance, we pick one of the candidates at random. We recalibrate the shipment quantity to reduce number of shipments to 1 for donor *i* using the following formulas (Eq. 4):

4 $$\begin{array}{l}\displaystyle{X}(i,j){\mathrm{max(new)}} = X(i,j){\mathrm{max(old)}} + {\sum} \,{{\mathrm{all}}\;{\mathrm{other}}\;{\mathrm{positive}}\;X(i,j)\,{\mathrm{values}}\;{\mathrm{for}}\;{\mathrm{donor}}\;i({\mathrm{old}})} \\ {\mathrm{all}}\;{\mathrm{other}}\;{\mathrm{positive}}\;X(i,j)\,{\mathrm{values}}\;{\mathrm{for}}\;{\mathrm{donor}}\;i({\mathrm{new}}) = 0\end{array}$$

**Fig. 5:**
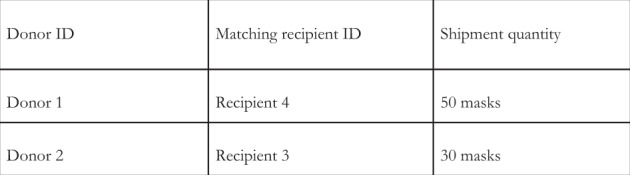
Algorithm generated donor–recipient matching table. Extracted values that are rendered back to the user in the form of a matching table.

Step 7: We extract final *X*(*i*,*j*) values and matches (*i*,*j*) and render them back to the user in the form of a matching table (Fig. 5).

Volunteers are given matching tables and coordinate with donors and recipients over email and/or phone to facilitate and confirm drop-off and delivery. After a volunteer verified that the donor still had the PPE donation to give, a donor was considered “matched” to the recipient facility and that “match” was documented. This project was undertaken as a Quality Improvement Initiative and as such was not formally supervised by the Institutional Review Board per author institutional policies.

### Reporting Summary

Further information on research design is available in the Nature Research Reporting Summary linked to this article.

## Supplementary information

Reporting Summary

## Data Availability

Due to the existence of sensitive data such as personal home addresses and individual provider PPE requests, the data are not publicly available. However, the algorithm inputs are standardized; external researchers can replicate our work by using the custom code available below and inputting data formatted with the table headings in Fig. 1. Please reach out to the corresponding author at Massachusetts General Hospital for more information.

## Code availability

The matching algorithm described in this study was implemented through custom code. This code is freely available at https://github.com/GetUsPPE/project_stanley.
