# Supplementary material for: Algorithmic matching of personal protective equipment donations with healthcare facilities during the COVID-19 pandemic
Source: NPJ Digit Med. 2021 Jan 29;4:13. doi: 10.1038/s41746-020-00375-3 (PMC7846564; doi:10.1038/s41746-020-00375-3)
Supplement: Supplementary file 1 — Reporting Summary [file 41746_2020_375_MOESM1_ESM.pdf]

nature research

Corresponding author(s): Shuhan He

Last updated by author(s): 2020/10/09

Reporting Summary

Nature Research wishes to improve the reproducibility of the work that we publish. This form provides structure for consistency and transparency in reporting. For further information on Nature Research policies, see our Editorial Policies and the Editorial Policy Checklist.

Statistics

For all statistical analyses, confirm that the following items are present in the figure legend, table legend, main text, or Methods section.

n/a	Confirmed	
☒	☐	The exact sample size (n) for each experimental group/condition, given as a discrete number and unit of measurement
☒	☐	A statement on whether measurements were taken from distinct samples or whether the same sample was measured repeatedly
☒	☐	The statistical test(s) used AND whether they are one- or two-sided<br>*Only common tests should be described solely by name; describe more complex techniques in the Methods section.*
☒	☐	A description of all covariates tested
☐	☒	A description of any assumptions or corrections, such as tests of normality and adjustment for multiple comparisons
☐	☒	A full description of the statistical parameters including central tendency (e.g. means) or other basic estimates (e.g. regression coefficient) AND variation (e.g. standard deviation) or associated estimates of uncertainty (e.g. confidence intervals)
☒	☐	For null hypothesis testing, the test statistic (e.g. F, t, r) with confidence intervals, effect sizes, degrees of freedom and P value noted<br>*Give P values as exact values whenever suitable.*
☒	☐	For Bayesian analysis, information on the choice of priors and Markov chain Monte Carlo settings
☒	☐	For hierarchical and complex designs, identification of the appropriate level for tests and full reporting of outcomes
☒	☐	Estimates of effect sizes (e.g. Cohen's d, Pearson's r), indicating how they were calculated

Our web collection on statistics for biologists contains articles on many of the points above.

Software and code

Policy information about availability of computer code

Data collection	Data was collected through the publicly available GetUsPPE.org platform. Parameters and data can be inputted at GetUsPPE.org
Data analysis	The matching algorithm described in this study was implemented through custom code. This code is freely available at https://github.com/RohitJacob/mask_match

For manuscripts utilizing custom algorithms or software that are central to the research but not yet described in published literature, software must be made available to editors and reviewers. We strongly encourage code deposition in a community repository (e.g. GitHub). See the Nature Research guidelines for submitting code & software for further information.

Data

Policy information about availability of data

All manuscripts must include a data availability statement. This statement should provide the following information, where applicable:

- Accession codes, unique identifiers, or web links for publicly available datasets
- A list of figures that have associated raw data
- A description of any restrictions on data availability

Due to the existence of sensitive data such as personal home addresses and individual provider PPE requests, the data are not publicly available. However, the algorithm inputs are standardized; external researchers can replicate our work by using the custom code available below and inputting data formatted with the table headings in Figure 1. Please reach out to the corresponding author at Massachusetts General Hospital for more information.

Field-specific reporting

Please select the one below that is the best fit for your research. If you are not sure, read the appropriate sections before making your selection.

☐ Life sciences ☒ Behavioural & social sciences ☐ Ecological, evolutionary & environmental sciences

For a reference copy of the document with all sections, see nature.com/documents/nr-reporting-summary-flat.pdf

Behavioural & social sciences study design

All studies must disclose on these points even when the disclosure is negative.

Study description	Mixed methods case study
Research sample	Our Research sample consisted of matching of 83,136 items of PPE to 135 healthcare facilities in need across the United States. Of these 135 donor-recipient matches, hospitals made up the largest proportion of healthcare facility recipients (27%), with surgical masks making up most of the matched donations (65%). Matched donation sizes varied, with the largest proportion being between 100-500 items (27%), followed by small donations of 10-50 and 0-10 items (both 24%).
Sampling strategy	Originally formed as a coalition through the viral Twitter hashtags #GetUsPPE and #GetMePPE GetUsPPE.org quickly became a nationwide organization focused on a mission to "build a national, centralized platform to enable communities to get PPE to healthcare providers on the frontlines of the COVID-19 pandemic." The original platform specification empowered donors with smaller quantities of PPE (e.g. several boxes of gloves, 3-4 packaged N95s) to facilitate their own donations by selecting an organization in need from a map that populated potential recipient organizations from the database. These requirements quickly changed due to the need for recipient organizations information to be protected given large amounts of PPE-related scams and reports of retribution by healthcare organizations toward employees who raised concerns about PPE shortages. Additionally, the wide variation among donations in terms of quantity, item type, and geography demonstrated a need for the organization to assume an active role in matching donors with the appropriate recipient. Thus both recipients and donors would input their information into the form found on GetUsPPE.org
Data collection	The data was collected on GetUsPPE.org with volunteer donors of PPE who indicated the quantity and types of PPE available. The recipients were institutions or individuals who requested PPE through the GetUsPPE.org database.
Timing	From April 14th to April 27th, 2020, we used the PPE matching algorithm to match 83,136 items of PPE to 135 healthcare facilities in need across the United States
Data exclusions	No data of PPE and matching was categorically excluded during this period.
Non-participation	none
Randomization	The treatment and control group were not randomized.

Reporting for specific materials, systems and methods

We require information from authors about some types of materials, experimental systems and methods used in many studies. Here, indicate whether each material, system or method listed is relevant to your study. If you are not sure if a list item applies to your research, read the appropriate section before selecting a response.

Materials & experimental systems

n/a	Involved in the study	
☒	☐	Antibodies
☒	☐	Eukaryotic cell lines
☒	☐	Palaeontology and archaeology
☒	☐	Animals and other organisms
☒	☐	Human research participants
☒	☐	Clinical data
☒	☐	Dual use research of concern

Methods

n/a	Involved in the study	
☒	☐	ChIP-seq
☒	☐	Flow cytometry
☒	☐	MRI-based neuroimaging
